# The antimicrobial activity of heterotrophic bacteria isolated from the marine sponge *Erylus deficiens* (Astrophorida, Geodiidae)

**DOI:** 10.3389/fmicb.2015.00389

**Published:** 2015-05-07

**Authors:** Ana Patrícia Graça, Flávia Viana, Joana Bondoso, Maria Inês Correia, Luis Gomes, Madalena Humanes, Alberto Reis, Joana R. Xavier, Helena Gaspar, Olga M. Lage

**Affiliations:** ^1^Department of Biology, Faculty of Sciences, University of PortoPorto, Portugal; ^2^Centre of Marine and Environmental Research (CIIMAR)Porto, Portugal; ^3^Centro de Química e Bioquímica e Departamento de Química e Bioquímica, Faculdade de Ciências, Universidade de LisboaLisboa, Portugal; ^4^Bioenergy Unit, National Laboratory for Energy and Geology I.P.Lisboa, Portugal; ^5^Department of Biology and Centre for Geobiology, University of BergenBergen, Norway

**Keywords:** sponge associated heterotrophic bacteria, *Erylus deficiens*, bioactivity screening assay, PKS-I and NRPS genes, *Candida albicans*

## Abstract

Interest in the study of marine sponges and their associated microbiome has increased both for ecological reasons and for their great biotechnological potential. In this work, heterotrophic bacteria associated with three specimens of the marine sponge *Erylus deficiens*, were isolated in pure culture, phylogenetically identified and screened for antimicrobial activity. The isolation of bacteria after an enrichment treatment in heterotrophic medium revealed diversity in bacterial composition with only *Pseudoalteromonas* being shared by two specimens. Of the 83 selected isolates, 58% belong to *Proteobacteria*, 23% to *Actinobacteria* and 19% to *Firmicutes*. Diffusion agar assays for bioactivity screening against four bacterial strains and one yeast, revealed that a high number of the isolated bacteria (68.7%) were active, particularly against *Candida albicans* and *Vibrio anguillarum*. *Pseudoalteromonas*, *Microbacterium*, and *Proteus* were the most bioactive genera. After this preliminary screening, the bioactive strains were further evaluated in liquid assays against *C. albicans*, *Bacillus subtilis* and *Escherichia coli*. Filtered culture medium and acetone extracts from three and 5 days-old cultures were assayed. High antifungal activity against *C. albicans* in both aqueous and acetone extracts as well as absence of activity against *B. subtilis* were confirmed. Higher levels of activity were obtained with the aqueous extracts when compared to the acetone extracts and differences were also observed between the 3 and 5 day-old extracts. Furthermore, a low number of active strains was observed against *E. coli*. Potential presence of type-I polyketide synthases (PKS-I) and non-ribosomal peptide synthetases (NRPSs) genes were detected in 17 and 30 isolates, respectively. The high levels of bioactivity and the likely presence of associated genes suggest that *Erylus deficiens* bacteria are potential sources of novel marine bioactive compounds.

## Introduction

Marine sponges (Phylum Porifera) are sessile filter-feeding organisms and represent a significant component of benthic communities' worldwide (Sarà and Vacelet, 1973). They harbor a large community of diverse microorganisms, representing up to 50–60% of the sponge biomass (Thomas et al., 2010), which develop symbiotic relationships essential to their biology. Sponges have been the focus of many studies as they have proved to be a rich source of biologically active secondary metabolites. These compounds may serve as a defense strategy to escape from predators, thus playing a crucial role in sponge survival in the marine ecosystem (Thakur and Müller, 2004). Since the discovery of spongothymidine and spongouridine in the early 50's (Bergmann and Feeney, 1950, 1951), a great number of biologically active compounds were isolated from marine sponges and their associated microorganisms (Taylor et al., 2007; Thomas et al., 2010). About 300 novel compounds were reported in 2011 from the phylum Porifera (Blunt et al., 2013) and more sponge-derived compounds are subjected to clinical and preclinical trials than from any other marine phylum (Martins et al., 2014). The chemical diversity of sponge-derived products is remarkable and their biological activity ranges from anti-inflammatory, antitumour, immuno- or neurosuppressive, antiviral, antimalarial, antibiotic to antifouling (Imhoff et al., 2011). However, many of the bioactive compounds found in sponges are, in fact, produced by their associated microbial communities (Wang, 2006; Khan et al., 2014). This is the case, for instance, of the peptide thiocoraline, produced by a sponge-associated *Micromonospora* species (Romero et al., 1997) and the cytotoxic and antibacterial tetrabromodiphenyl ethers produced by a *Vibrio* spp. associated with the sponge *Dysidea* sp. (Elyakov et al., 1991). This microbial community represents a treasure trove of novel molecules for marine biotechnology. Due to the increasing resistance of bacteria against the common antibiotics there is still a pressing need to find new drugs (Bull and Stach, 2007; Kumarasamy et al., 2010). By the end of 2008, about 3000 natural products had been identified from marine microorganisms (Laatsch, 2008). The most relevant phyla of bacterial producers of new compounds are *Actinobacteria* (40%), *Cyanobacteria* (33%), *Proteobacteria* (12%), and *Firmicutes* and *Bacteroidetes* (5% each) (Williams, 2009). In addition to these, archaea, fungi, unicellular algae and other bacterial phyla namely *Acidobacteria*, *Chloroflexi*, *Deinococcus*-*Thermus*, *Firmicutes*, *Gemmatimonadetes*, *Nitrospira*, *Planctomycetes*, *Poribacteria*, *Spirochaetes*, and *Verrucomicrobia*, are also present in the sponge microbial community as revealed mainly by culture-independent molecular methods (Taylor et al., 2007).

In a genomic era, it is easy to assess by molecular methods the bacterial potential for the production of bioactive molecules. These natural bioactive secondary metabolites can be the result of the activity of non-ribosomal peptide synthetases (NRPSs) and polyketide synthases (PKSs) that are large multi-modular, multi-domain enzymes. The minimal set of domains that have to be present in these biosynthetic pathways are ketosynthase (KS), acyltransferase (AT) and acyl carrier proteins in PKSs (Khan et al., 2014) and adenylation (A), condensation (C) and peptidyl carrier proteins (PCPs) for peptide elongation in NRPSs (Jenke-Kodama and Dittmann, 2009). In addition to the synthesis of these defined domains, optional domains can also be produced like ketoreductase. The intra and inter variability of the enzymatic modules codified by PKS and NRPS genes results in the production of diverse bioactive compounds. Antibiotic molecules such erythromycin, tetracycline and glycopeptides of the vancomycin family were reported to have origin in PKS and NRPS productions (Fischbach and Walsh, 2006). In fact, many of the complex polyketides isolated from sponges are the most promising compounds with pharmacological application (Hochmuth and Piel, 2009). Therefore, the search for the genes responsible for the expression of these enzymes is indicative of the biotechnological potential of a specific organism.

The marine sponge genus *Erylus*, Gray, 1867, contains approximately 70 species and is known to inhabit the Mediterranean Sea, and the Atlantic and Pacific Oceans (Adams and Hooper, 2001). Species of this genus have been reported to possess characteristic bioactive glycosides named, according to their aglycone skeleton, erylosides or erylusamines (Kalinin et al., 2012). Some of these glycolipids have been shown to inhibit tumor cells, fungi, bacteria, viruses including the human immunodeficiency virus (HIV), neuraminidase, interleukin-6, thrombin receptor antagonist, and *in vitro* human platelet aggregation (Carmely et al., 1989; Fusetani et al., 1993; Gulavita et al., 1994; Sata et al., 1994; Stead et al., 2000; Kubanek and Fenical, 2001; Shin et al., 2001; Takada et al., 2002; Van Altena et al., 2003; Fouad et al., 2004; Sandler et al., 2005; Okada et al., 2006; Afiyatullov et al., 2007; Antonov et al., 2007). Furthermore, *Erylus* spp. are well known producers of oligoglycosides with antifeedant activity involved in chemical defenses of sponges against predatory fish (Kubanek et al., 2000; Kubanek and Fenical, 2001; Antonov et al., 2011) and with larvicidal activity (Carballeira and Negrón, 1991). Crude extracts of *Erylus deficiens* from the Portuguese coast were found to possess indoleamine 2,3-dioxygenase (IDO) inhibitory activity (Gaspar et al., 2012). This enzyme plays a fundamental role in the kynurenine pathway, one of the major tryptophan catabolism routes with implications in cancer and in CNS disorders like Alzheimer's disease and cerebral malaria (Chen and Guillemin, 2009). The bioactive potential of bacteria associated with *Erylus discophorus* was evidenced by Graça et al. (2013). The work showed that 31% of the isolated bacteria were able to produce antimicrobial metabolites being this the only study performed with bacteria associated with the *Erylus* sponges. This lack of results associated with the low yield of metabolites normally originated from marine sponges, together with the importance of obtaining new bioactive bacteria to avoid the undesired exploitation of natural sponges, lead us to assess in three specimens of *E. deficiens* collected in the Gorringe Bank, an isolated seamount in the Atlantic, the potential of heterotrophic culturable bacteria as actual producers of antimicrobial compounds by screening and molecular assays. As glycolipids from these sponges are already being studied by others, NRPS and PKS appeared as good gene targets for the search of new potential bioactivities.

## Materials and methods

### Sponge sampling and identification

Three specimens of *E*. *deficiens* Topsent, 1927 (Demospongiae, Astrophorida, Geodiidae) were collected by scuba diving at approximately 38 m depth on Gettysburg (specimens #66 and #91—lat. 36° 31′ 10″ N, long. 11° 34′ 10″ W) and Ormonde (specimen #118—lat. 36° 42′ 70″ N, 11° 09′ 70″ W) Peaks, in the course of LusoExpedição Olympus 2008 to the Gorringe Bank, a large seamount located 150 Km off the southwest coast of Portugal. The samples were placed individually in ziploc® plastic bags and then carried to the laboratory for identification and microbial isolation. Voucher samples were preserved in 90% ethanol for taxonomic identification and deposited in the Biology Department's zoological collection of the University of the Azores (collection DBUA.Por). Specimens were identified using general external and internal morphological characters analysis, i.e., shape, type, size and arrangement of skeletal structures (spicules) following the Systema Porifera classification system (Hooper and Van Soest, 2002). This species was previously reported in this seamount and identified as *Erylus* sp. (Xavier and Van Soest, 2007) and further examination of additional samples, including specimens from Madeira Island, the type-locality of *E. deficiens*, enabled its identification to species level.

### Heterotrophic bacterial isolation

Fragments of sponge tissues were placed under aseptic conditions in sterile Erlenmeyers containing M514 medium (Bacto-peptone (Difco) 5 g/L; yeast Extract (Oxoid) 1 g/L and sea salts (Red Sea®) 36 g/L). This enrichment was performed to promote faster growing and abundant heterotrophic bacteria. When noticeable bacterial growth occurred, usually after 1 week without stirring, several dilutions (10^−1^, 10^−6^, and 10^−12^) were prepared and, 100 μL of each, spread in several solid marine media prepared with 0.2 μm filtered natural seawater and 1.8% agar: modified M13 (Lage and Bondoso, 2011), PYGV (0.025% peptone, 0.025% yeast extract, 0.025% glucose, 10 mL.L^−1^ vitamin solution n°6, 20 mL.L^−1^ Hutner's basal salts (Cohen-Bazire et al., 1957), Marine Agar (MA - Difco), M17 (Difco), Glycerol Asparagine (1% glycerol, 0.1% L-asparagine, 0.1% K_2_HPO_4_), M4 (Zhang et al., 2006). Cultures were incubated in the dark at 26°C. Growth was monitored daily and distinct colony morphotypes were transferred to fresh medium for isolation. Pure bacterial cultures were cryopreserved in seawater supplemented with 20% glycerol at −80°C.

### Molecular identification of isolates

Isolates were identified by 16S rRNA gene sequence analysis after amplification with the primers 27f and 1492r (Lane, 1991). The PCR was carried out as described by Bondoso et al. (2011), using, as template, material from a single colony or genomic DNA extracted with the Bacterial DNA Isolation Kit (Omega), according to the manufacturer' instructions. PCR products were directly sequenced at Macrogen Europe (www.Macrogen.com).

The obtained 16S rRNA gene sequences were processed with the Sequencing Analysis 5.2 (Applied Biosystems) and assembled with Vector NTI Advance™ 10.3. The resulting 16S rRNA gene sequences were then blasted on SeqMatch (Ribossomal Database Project) (Cole et al., 2014) against the nucleotide database of GenBank (NCBI) and the closest neighbors were downloaded. The sequences were corrected manually using the alignment generated. Subsquently, sequences with 818 bp were used for molecular evolutionary analyses in MEGA version 5.2 (Tamura et al., 2011). A final maximum likelihood (ML) phylogenetic tree was generated applying General Time Reversible model and Gamma distributed with Invariant sites (G+I). Validation of reproducibility of the branching patterns was made by bootstrap based on 1000 resamplings. Pairwise sequence similarities based on Jukes-Cantor model were calculated in MEGA. Alignment gaps and missing data were not included in the calculations. Different phylotypes were considered based on a 97% 16S rRNA gene threshold (Stackebrandt and Goebel, 1994). The sequences of the isolated bacteria used in this study were deposited in GenBank with the serial accession numbers KP120772- KP120854.

### Bioactivity of the bacterial isolates

To assess the antimicrobial bioactivity of the sponge-isolated bacteria, designated hereunder as test bacteria, a simple co-culture agar diffusion assay (pre-screening) was performed against a panel of target organisms. This panel was constituted by Gram negative and positive bacteria commonly used in bioactivity bioassays, a relevant environmental species and a human pathogenic yeast. Thus, the bioactivity against four bacterial strains (*Bacillus subtilis* ATCC 6633*, B. cereus* FCUP collection*, Escherichia coli* ATCC 25922 and the fish pathogen *Vibrio anguillarum* FCUP collection) and a yeast (clinical isolate of *Candida albicans*) was evaluated. The agar diffusion assay was performed in MA medium, solidified with 1.4% agar as this medium allowed the simultaneous growth of the test bacteria and the target microorganisms. The test bacteria were heavily inoculated on half a Petri dish containing MA medium and incubated vertically to favor the diffusion of the metabolites to the non-inoculated part of the medium, at 26°C for 48 h. By this time, the fast growing test bacteria attained the stationary growth phase. Subsequently, the five target microorganisms were inoculated on the other half by streaking a line perpendicularly to the test bacteria and parallel to each other. The cultures were grown at 37 °C in the same position as described before. The presence of growth inhibition was observed after 24 h. Target organisms grown in MA medium were used as controls.

To confirm the positive results obtained with the preliminary assay, a liquid screening assay was performed based on Graça et al. (2013). Of the 57 bacteria that showed antimicrobial activity only 48 were assayed as nine lost viability (#91_37, #91_40, #91_43, #91_45, #118_14, #118_23, #118_27, #118_33, and #118_39). The bacteria were grown in MB for 3 and 5 days at 25°C and 200 r.p.m. At days 3 and 5, an aliquot of 2 mL of the culture was centrifuged and the supernatant was 0.22 μm filtered and stored at −20°C—aqueous extract. Another aliquot of 2 mL of culture was mixed with 1.8 mL acetone +0.2 mL DMSO, incubated for 1 h after which the upper phase was collected and stored at −20°C—acetone extract. In 96-well plates, duplicates of 10 μL of each extract were assayed against 90 μL of each target microorganism cultivated in LB medium (≈ 2.5 × 10^5^ CFUs/mL). Additionally, 100 μL of each target microorganism were cultivated to allow the assessment of the total growth of the microorganisms. As positive controls, amphotericin B (0.19; 0.39; 0.78; and 1.56 μg/ml) and rifampicin (62.5; 125; 250; and 500 mg/ml) were used against *C. albicans* and *E. coli*, respectively. LB medium was used as negative control. In order to assess the possible interference of the solvents acetone+DMSO present in the extracts, both *C. albicans* and *E. coli* were grown in the presence of the same concentration used for the bioactivity assays. As no significant variation was induced by the solvents used to obtain the extracts, these values will not be considered in the calculation of bioactivities. The plates were incubated at 37°C for 24 h at 250 r.p.m. The optical density (OD) of the cultures was measured at 600 nm in a Multiskan GO plate reader (Thermo Scientific). The growth of the target microorganisms (%) was calculated by the following equation: (OD_cultures with extract_ – OD_LB medium_)/OD_positive control_ × 100. Variability in the positive controls was of about 10%, reason why only growth inhibitions of equal or more than 25% were considered.

### Search for PKS-I and NRPS genes

The presence of genes involved in the production of secondary metabolites was screened in all the isolates obtained. The degenerate primers MDPQQRf (5′-RTRGAYCCNCAGCAICG-3′) and HGTGTr (5′-VGTNCCNGTGCCRTG-3′) (Kim et al., 2005) were used to amplify the β-ketosynthase (KS) domain fragment within the Type I polyketide synthase genes PKS-I. For the amplification of NRPS, primers MTF2 [5′- GCNGG(C/T)GG(C/T)GCNTA(C/T)GTNCC-3′(AGGAYVP, core motif I)] and MTR [5′- CCNCG(AGT)AT(TC)TTNAC(T/C)TG-3′(QVKIRG, core motif V)] (Neilan et al., 1999) were used. The PCR mixture contained 1x PCR buffer, 0.8 units of Go Taq DNA Polymerase (Promega), 0.2 mM of each dNTPs, 0.1 μM of each primer and 1 μL genomic DNA as template. The PCR profile consisted of an initial denaturing step of 5 min at 95°C, 11 cycles of 1 min at 95°C, 30 s at 60°C and 1 min at 72°C, with the annealing temperature reduced by 2°C per cycle, followed by 30 cycles of 95°C for 1 min, 40°C for 30 s and 72°C for 1 min with a final extension of 10 min at 72°C (Kim et al., 2005). The PCR was carried out in a MyCycler™ Thermo Cycler (Bio-Rad) and the amplicons were visualized in a Roti-Safe (Roth) stained 1.2% agarose gel.

## Results

### 16S rRNA gene analysis identification

**Figure 1:**
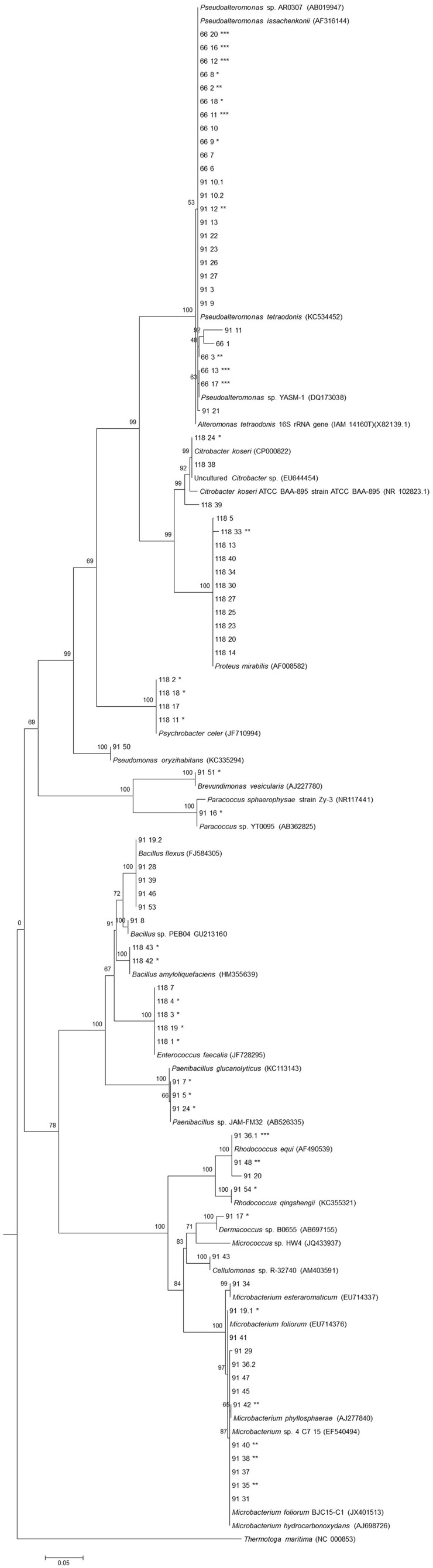
**Phylogenetic 16S rRNA gene tree generated by maximum-likelihood analysis based in General Time Reversible model and Gamma distributed with Invariant sites (G+I) indicating the relationship of the heterotrophic bacteria isolated from three *E. deficiens* specimens**. *Thermatoga maritima* was used as outgroup. Bar – 0.05 substitutions per 100 nucleotides. ^*^Presence of NRPS genes; ^**^Presence of PKS-I genes; ^***^Presence of both genes.

A total of 83 isolates were selected after enrichment procedure from the three specimens of *E. deficiens*. All assayed media allowed the isolation of bacterial strains although marine agar was the medium that provided the highest number of isolates and broad diversity from the three sponges. Curiously, M17 medium that is designed to grow fastidious lactic streptococci, allowed the growth of several *Firmicutes* and *Actinobacteria*. The majority of the strains obtained in this study after the enrichment belong to *Proteobacteria* (58%), followed by *Actinobacteria* (23%) and *Firmicutes* (19%) (Table 1; Figure 1). Within the *Proteobacteria*, the most abundant class was the *Gammaproteobacteria* with 46 isolates and the genus *Pseudoalteromonas* (27 isolates). Within the *Actinobacteria*, the genus *Microbacterium* was represented by 13 isolates. Of the selected strains and based on a 16S rRNA gene 97% threshold, 10 different phylotypes were from sponge #91, five phylotypes from sponge #118 and only one phylotype from sponge #66.

**Table 1:** **Affiliation, bioactivity and presence of the PKS-I and NRPS genes of *E*. *deficiens* bacterial isolates**.

**Isolate**	**Similarity^a^**	**Related strain^a^**	**Phylum/Class**	**Genera**	**Activities in solid assays**	**Activities in liquid assays**	**PKS-I**	**NRPS**
#91_43	99.8	*Cellulomonas* sp. R-32740; AM403591	*Actinobacteria*	*Cellulomonas*	CA, VA	ND	ND	ND
#91_17	98.9	*Dermacoccus* sp. Ellin185; AF409027	*Actinobacteria*	*Dermacoccus*	CA, VA	N	ND	D
#91_34	100	*Microbacterium esteraromaticum*; 2122; EU714337	*Actinobacteria*	*Microbacterium*	CA	CA	ND	ND
#91_42	99.9	*Microbacterium foliorum*; 150; EU714333	*Actinobacteria*	*Microbacterium*	N		D	ND
#91_19.1	99.8	*Microbacterium foliorum*; 720; EU714376	*Actinobacteria*	*Microbacterium*	N		ND	D
#91_29	99.9	*Microbacterium foliorum*; Bjc15-C14; JX464206	*Actinobacteria*	*Microbacterium*	CA	CA	ND	ND
#91_36.2	99.2	*Microbacterium foliorum*; BJC15-C1; JX401513	*Actinobacteria*	*Microbacterium*	CA, VA	CA	ND	ND
#91_37	100	*Microbacterium foliorum*; BJC15-C1; JX401513	*Actinobacteria*	*Microbacterium*	CA, BS, VA	ND	ND	ND
#91_40	99.4	*Microbacterium foliorum*; Bjc15-C14; JX464206	*Actinobacteria*	*Microbacterium*	CA, VA	ND	D	ND
#91_41	99.4	*Microbacterium foliorum*; S2-157; JQ660074	*Actinobacteria*	*Microbacterium*	N		ND	ND
#91_31	99.9	*Microbacterium hydrocarbonoxydans*; 3084; EU714352	*Actinobacteria*	*Microbacterium*	CA	CA	ND	ND
#91_38	99.7	*Microbacterium hydrocarbonoxydans*; 3517; EU714368	*Actinobacteria*	*Microbacterium*	N		D	ND
#91_45	99.7	*Microbacterium hydrocarbonoxydans*; 3517; EU714368	*Actinobacteria*	*Microbacterium*	CA	ND	ND	ND
#91_47	99.7	*Microbacterium hydrocarbonoxydans*; HR73; JF700446	*Actinobacteria*	*Microbacterium*	N		ND	ND
#91_35	99.8	*Microbacterium phyllosphaerae* (T); DSM 13468; P 369/06; AJ277840	*Actinobacteria*	*Microbacterium*	CA, BS, VA	CA	D	ND
#91_20	97.0	*Rhodococcus hoagii*; CUB1156; AJ272469	*Actinobacteria*	*Rhodococcus*	N		ND	ND
#91_36.1	100	*Rhodococcus equi*; type strain: DSM20307; X80614	*Actinobacteria*	*Rhodococcus*	EC, CA, VA	N	D	D
#91_48	99.9	*Rhodococcus hoagii; type strain:* DSM20307; X80614	*Actinobacteria*	*Rhodococcus*	N		D	ND
#91_54	99.9	*Rhodococcus qingshengii*; KUDC1814; KC355321	*Actinobacteria*	*Rhodococcus*	CA, VA	CA	ND	D
#91_51	99.9	*Brevundimonas vesicularis* (T); LMG 2350 (T); AJ227780	*Alphaproteobacteria*	*Brevundimonas*	N		ND	D
#91_16	99.0	*Paracoccus sphaerophysae strain Zy-3*; NR117441	*Alphaproteobacteria*	*Paracoccus*	CA, EC	CA	ND	D
#118_42	100	*Bacillus amyloliquefaciens*; BAC3048; HM355639	*Firmicutes*	*Bacillus*	N		ND	D
#118_43	100	*Bacillus amyloliquefaciens*; BAC3048; HM355639	*Firmicutes*	*Bacillus*	N		ND	D
#91_46	100	*Bacillus aryabhattai*; D27; FR750269	*Firmicutes*	*Bacillus*	N		ND	ND
#91_19.2	100	*Bacillus flexus*; FJ584305	*Firmicutes*	*Bacillus*	N		ND	ND
#91_39	100	*Bacillus flexus*; FJ584305	*Firmicutes*	*Bacillus*	CA, VA	CA	ND	ND
#91_53	99.8	*Bacillus* sp. CCBAU 10722; EF377323	*Firmicutes*	*Bacillus*	CA, VA	N	ND	ND
#91_28	99.9	*Bacillus* sp. G2DM-19; DQ416802	*Firmicutes*	*Bacillus*	N		ND	ND
#91_8	99.7	*Bacillus* sp. PEB04; GU213160	*Firmicutes*	*Bacillus*	CA	CA	ND	ND
#118_1	100	*Enterococcus* sp. T004_8; JQ739635	*Firmicutes*	*Enterococcus*	N		ND	D
#118_4	99.8	*Enterococcus faecalis*; AF039902	*Firmicutes*	*Enterococcus*	VA	CA	ND	D
#118_3	99.9	*Enterococcus faecalis*; GIMC503:NBS-2; JF728295	*Firmicutes*	*Enterococcus*	CA, VA	CA	ND	D
#118_19	99.9	*Enterococcus faecalis*; Y18293	*Firmicutes*	*Enterococcus*	EC, CA, VA	CA	ND	D
#118_7	100	*Enterococcus faecalis*; ZZ18; HM776212	*Firmicutes*	*Enterococcus*	VA	CA	ND	ND
#91_5	99.8	*Paenibacillus glucanolyticus*; KSI 1323; KC113143	*Firmicutes*	*Paenibacillus*	N		ND	D
#91_7	99.8	*Paenibacillus* sp. JAM-FM32; AB526335	*Firmicutes*	*Paenibacillus*	N		ND	D
#91_24	100	*Paenibacillus* sp. JAM-FM32; AB526335	*Firmicutes*	*Paenibacillus*	CA, VA	CA	ND	D
#118_24	98.2	*Citrobacter koseri* ATCC BAA-895; CP000822	*Gammaproteobacteria*	*Citrobacter*	N		ND	D
#118_38	99.7	*Citrobacter koseri* ATCC BAA-895; CP000822	*Gammaproteobacteria*	*Citrobacter*	N		ND	ND
#118_39	95.0	*Citrobacter koseri* ATCC BAA-895; CP000822	*Gammaproteobacteria*	*Citrobacter*	CA	ND	ND	ND
#118_20	99.7	bacterium NLAE-zl-H279; JX006497	*Gammaproteobacteria*	*Proteus*	EC, CA	CA	ND	ND
#118_13	99.9	*Proteus mirabilis*; ATCC 29906T; AF008582	*Gammaproteobacteria*	*Proteus*	CA	EC, CA	ND	D
#118_14	99.6	*Proteus mirabilis*; ATCC 29906T; AF008582	*Gammaproteobacteria*	*Proteus*	EC, CA, VA	ND	ND	—
#118_23	99.7	*Proteus mirabilis*; ATCC 29906T; AF008582	*Gammaproteobacteria*	*Proteus*	CA	ND	ND	ND
#118_27	100	*Proteus mirabilis*; ATCC 29906T; AF008582	*Gammaproteobacteria*	*Proteus*	CA	ND	ND	ND
#118_30	100	*Proteus mirabilis*; ATCC 29906T; AF008582	*Gammaproteobacteria*	*Proteus*	N		ND	ND
#118_34	100	*Proteus mirabilis*; ATCC 29906T; AF008582	*Gammaproteobacteria*	*Proteus*	N		ND	ND
#118_40	99.9	*Proteus mirabilis*; ATCC 29906T; AF008582	*Gammaproteobacteria*	*Proteus*	N		ND	ND
#118_5	99.7	*Proteus mirabilis*; IFS10; AB272366	*Gammaproteobacteria*	*Proteus*	CA	CA	ND	ND
#118_25	99.8	*Proteus mirabilis*; LH-52; JN861767	*Gammaproteobacteria*	*Proteus*	N		ND	ND
#118_33	99.3	*Proteus mirabilis*; LH-52; JN861767	*Gammaproteobacteria*	*Proteus*	CA	ND	D	ND
#66_2	97.9	Bacterium K2-82; AY345483	*Gammaproteobacteria*	*Pseudoalteromonas*	N		D	ND
#91_3	99.9	Bacterium Antarctica-11; EF667985	*Gammaproteobacteria*	*Pseudoalteromonas*	CA, VA	CA	ND	ND
#91_9	99.9	Bacterium Antarctica-11; EF667985	*Gammaproteobacteria*	*Pseudoalteromonas*	CA	CA	ND	ND
#91_12	99.9	Bacterium Antarctica-11; EF667985	*Gammaproteobacteria*	*Pseudoalteromonas*	CA, VA	CA	D	ND
#91_13	99.9	Bacterium Antarctica-11; EF667985	*Gammaproteobacteria*	*Pseudoalteromonas*	EC, CA, VA	CA	ND	ND
#91_23	99.9	Bacterium Antarctica-11; EF667985	*Gammaproteobacteria*	*Pseudoalteromonas*	CA	CA	ND	ND
#66_11	99.3	*Pseudoalteromonas issachenkonii* (T); KMM 3549; F13; AF316144	*Gammaproteobacteria*	*Pseudoalteromonas*	CA, VA	EC, CA	D	D
#91_22	98.6	*Pseudoalteromonas* sp. 12; DQ642815	*Gammaproteobacteria*	*Pseudoalteromonas*	CA, VA	CA	ND	ND
#66_17	100	*Pseudoalteromonas* sp. 8007; AM111068	*Gammaproteobacteria*	*Pseudoalteromonas*	EC, CA, VA	CA	D	D
#66_6	99.8	*Pseudoalteromonas* sp.; AR0307; AB019947	*Gammaproteobacteria*	*Pseudoalteromonas*	CA, VA	EC, CA	ND	ND
#66_7	99.8	*Pseudoalteromonas* sp.; AR0307; AB019947	*Gammaproteobacteria*	*Pseudoalteromonas*	CA, VA	CA	ND	ND
#66_16	99.9	*Pseudoalteromonas* sp.; AR0307; AB019947	*Gammaproteobacteria*	*Pseudoalteromonas*	N		D	D
#91_10.1	99.9	*Pseudoalteromonas* sp.; AR0307; AB019947	*Gammaproteobacteria*	*Pseudoalteromonas*	CA	EC, CA	ND	ND
#91_10.2	99.9	*Pseudoalteromonas* sp.; AR0307; AB019947	*Gammaproteobacteria*	*Pseudoalteromonas*	EC, CA, VA	CA	ND	ND
#66_10	98.6	*Pseudoalteromonas* sp. ARCTIC-P16; AY573035	*Gammaproteobacteria*	*Pseudoalteromonas*	VA	CA	D	D
#66_1	96.4	*Pseudoalteromonas* sp. BSi20325; DQ520886	*Gammaproteobacteria*	*Pseudoalteromonas*	CA	N	ND	ND
#66_8	99.6	*Pseudoalteromonas* sp. BSi20325; DQ520886	*Gammaproteobacteria*	*Pseudoalteromonas*	VA	CA	ND	D
#66_18	97.9	*Pseudoalteromonas* sp. BSi20325; DQ520886	*Gammaproteobacteria*	*Pseudoalteromonas*	N		ND	D
#91_26	98.0	*Pseudoalteromonas* sp. P1; EF627987	*Gammaproteobacteria*	*Pseudoalteromonas*	EC	CA	ND	ND
#66_20	98.9	*Pseudoalteromonas* sp. SM9913; CP001796	*Gammaproteobacteria*	*Pseudoalteromonas*	CA, VA	EC, CA	D	D
#66_13	100	*Pseudoalteromonas* sp. YASM-1; DQ173038	*Gammaproteobacteria*	*Pseudoalteromonas*	CA, VA	CA	D	D
#91_21	97.0	*Pseudoalteromonas tetraodonis*; Do-17; AB257325	*Gammaproteobacteria*	*Pseudoalteromonas*	CA	CA	ND	ND
#66_12	98.4	*Pseudoalteromonas tetraodonis;* SSA930; KC534452	*Gammaproteobacteria*	*Pseudoalteromonas*	CA, VA	CA	D	D
#66_3	97.5	*Pseudoalteromonas* sp. LOB-15; DQ412067	*Gammaproteobacteria*	*Pseudoalteromonas*	CA, VA	CA	D	ND
#91_11	95.2	Uncultured bacterium; 11A-6; FJ998316	*Gammaproteobacteria*	*Pseudoalteromonas*	CA	CA	ND	ND
#91_27	96.6	Uncultured *Pseudoalteromonas* sp.; DVBSW_J357; KF722314	*Gammaproteobacteria*	*Pseudoalteromonas*	CA	EC	ND	ND
#66_9	98.9	Uncultured *Pseudoalteromonas* sp.; CI66; FJ695586	*Gammaproteobacteria*	*Pseudoalteromonas*	CA, VA	EC, CA	ND	D
#91_50	100	*Pseudomonas oryzihabitans*; SFK5; KC335294	*Gammaproteobacteria*	*Pseudomonas*	CA, VA	EC, CA	ND	ND
#118_11	100	*Psychrobacter celer*; B_IV_3L25; JF710994	*Gammaproteobacteria*	*Psychrobacter*	VA	CA	ND	D
#118_2	99.8	*Psychrobacter celer*; 91; JF710993	*Gammaproteobacteria*	*Psychrobacter*	VA	CA	ND	D
#118_17	99.9	*Psychrobacter celer*; 91; JF710993	*Gammaproteobacteria*	*Psychrobacter*	EC, CA, VA	CA	ND	ND
#118_18	99.9	*Psychrobacter* sp. *2CpBB14; JN602232*	*Gammaproteobacteria*	*Psychrobacter*	N		ND	D

*CA, Candida albicans; EC, Escherichia coli; VA, Vibrio anguillarum; BS, Bacillus subtilis; N, no activity; D, detected; ND, not detected*.

a *Based on SeqMatch (Ribossomal Database Project) (Cole et al., 2014)*.

### Bioactivity of the bacterial isolates

**Figure 2:**
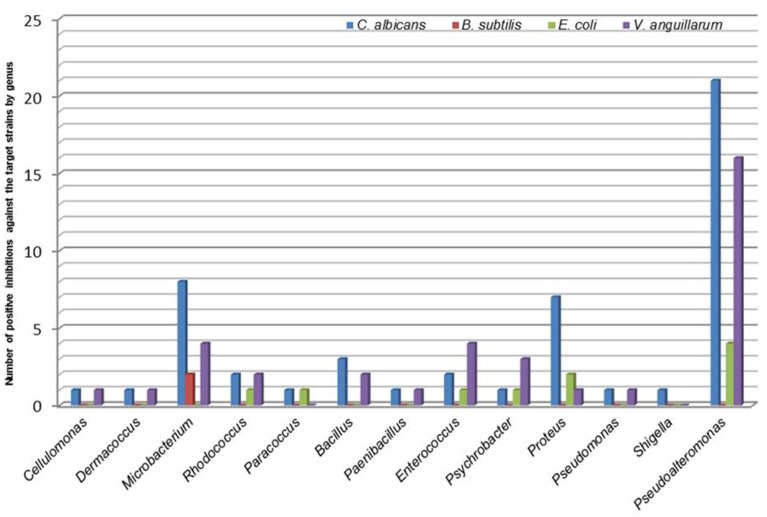
**Relation between the hits of bioactive genera and the target organisms studied in the preliminary screening**.

Through the co-culture diffusion pre-screening assay, 68.7% (57 bacteria) of the isolated bacteria showed antimicrobial activity against one or more of the target microorganisms tested. The highest number of positive hits were found in *Gammaproteobacteria* (63.2%) and *Actinobacteria* (21.1%) followed by *Firmicutes* (14.0%) and *Alphaproteobacteria* (1.8%). Of the thirteen genera showing antimicrobial bioactivity, *Pseudoalteromonas* (42.1%), *Microbacterium* (14.0%) and *Proteus* (12.3%) presented the highest positive hits. The analysis of bioactivity by test bacteria (Table 1) revealed a high number of strains with antifungal activity against *C. albicans* (87.7%—50 bacteria) and antibiotic against *V. anguillarum* (63.2%—36 bacteria) and, in lower number, against *E. coli* (17.5%—10 bacteria) *and B. subtilis* (3.5%—2 bacteria). None of the isolates produced effective bioactive compounds against the Gram positive *B. cereus*. Some bioactive bacteria were able to produce secondary bioactive metabolites against two or more target microorganisms. *Microbacterium* was the only genus able to produce bioactive compounds effective against *B. subtilis* (Figure 2).

**Figure 3:**
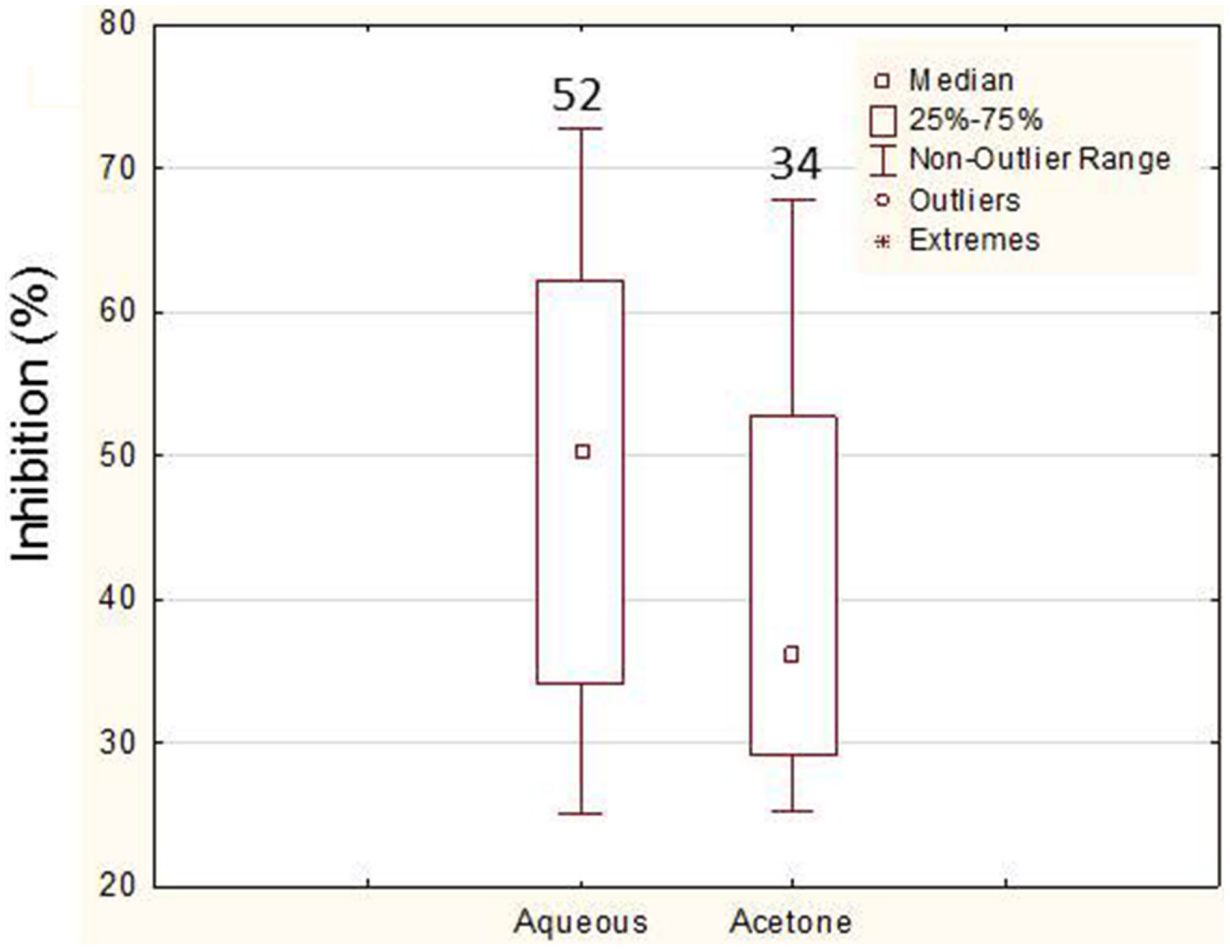
**Comparison of the levels of inhibition (higher than 25%) between the aqueous and the acetone extracts from *Erylus deficiens* isolates in the liquid assays**. The numbers above each box represent the number of bioactive extracts.

**Figure 4:**
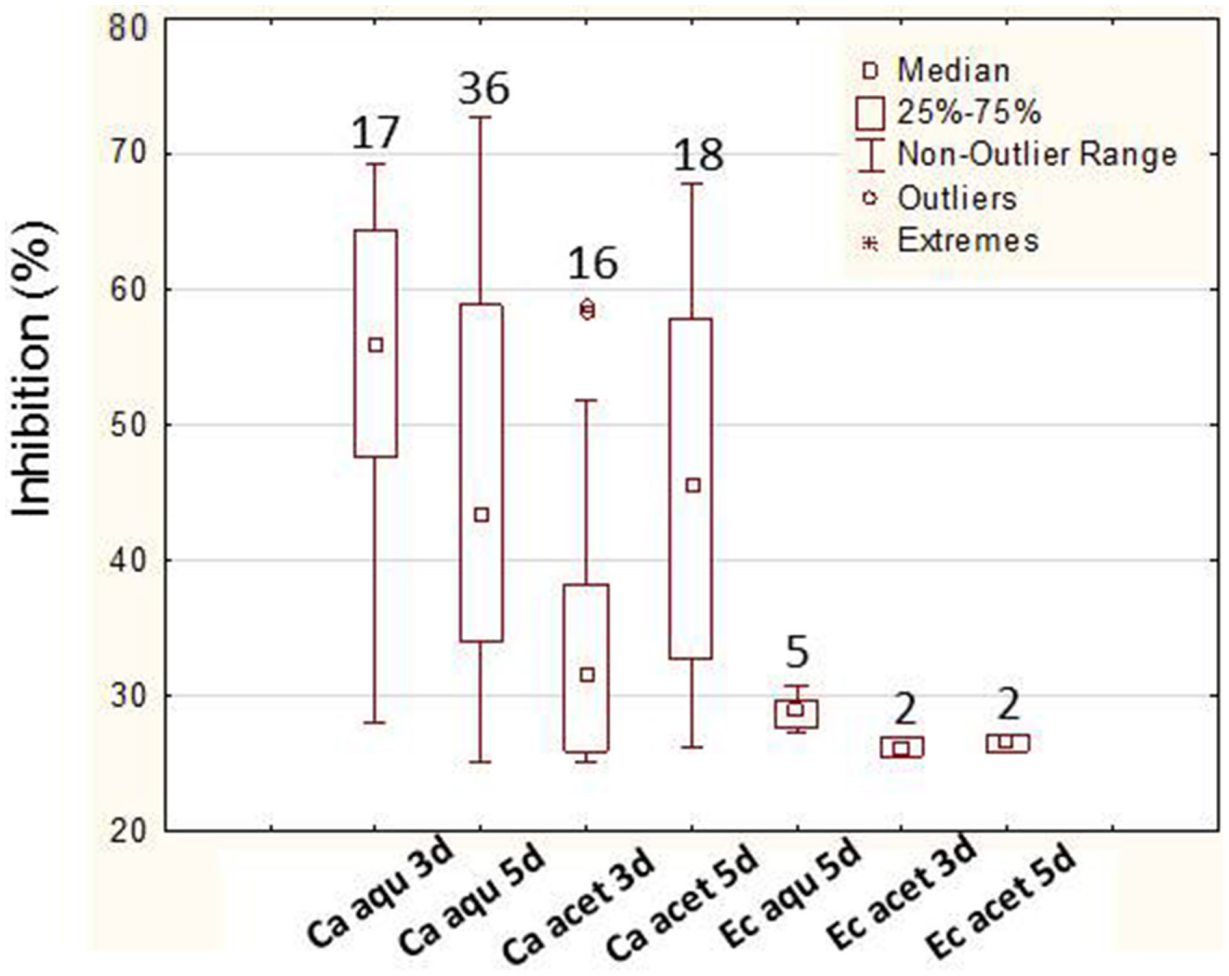
**Levels of inhibition (higher than 25%) of 3 and 5 days and aqueous (aqu) and acetone (acet) extracts from *Erylus deficiens* isolates against *C. albicans* (Ca) and *E. coli* (Ec)**. No result is shown for aqueous, 3 days extracts against *E. coli* due to the absence of inhibition. The numbers above each box represent the number of bioactive extracts.

In order to confirm the positive results obtained in the preliminary assay, a liquid screening assay was performed which allowed a more precise evaluation of the bioactivities. In this screening, 92% (45 bacteria) of the bacteria assayed showed bioactivity. Again, more bacteria showed bioactivity against *C. albicans* (80%—36 bacteria), only 4% (2 bacteria) showed activity against *E. coli* and 16% (7 bacteria) against both. A total of 86.6% bacteria (*n* = 39) showed activity in their aqueous extracts (87% against *C. albicans* and 13% against both *C. albicans* and *E. coli*) while only 62.2% bacteria (*n* = 28) showed bioactivity in acetone extracts. The levels of inhibition obtained (maximum value and median) with the aqueous extracts (3 and 5 days) were higher than the ones with acetone (Figure 3). The analysis of the bioactive extracts also showed higher levels of inhibition against *C. albicans* than against *E. coli* in which inhibition levels were very low (Figure 4). Furthermore, it was possible to verify in the assays against *C. albicans* that the 3 days acetone extracts were less effective than the aqueous and 5 days extracts. Although the median of 5 days aqueous extracts was not so high comparatively to the 3 days aqueous or the 5 days acetone extracts, the maximum value as well as the number of bioactive extracts were higher. No activity was obtained with 3 days aqueous extracts against *E. coli*.

### Search for PKS-I and NRPS genes

The β-ketosynthase domain fragment within the Type I polyketide synthase genes PKS-I was potentially amplified from 17 *E*. *deficiens* isolates (Table 1). Ten bacteria phylogenetically affiliated to *Pseudoalteromonas* from sponges #91 and #66, four to *Microbacterium* and two to *Rhodococcus* from sponge #91 and one to *Proteus* from sponge #118 amplified probable PKS-I sequences. The potential amplification of the motifs I and V of non-peptide synthetase adenylate-forming enzymes was observed in thirty strains (Table 1)—10 *Pseudoalteromonas* from sponge #66, four *Enterococcus* from sponge #118, three *Paenibacillus* from sponge #91, three *Psycrobacter* from sponge #118, two *Bacillus* from sponge #118, two *Rhodococcus* from sponge #91 and one *Dermacoccus*, one *Microbacterium*, one *Brevundimonas* and one *Paracoccus*, from sponge #91 and one *Citrobacter* and *Proteus* from sponge #118. A relatively high number of the bioactive isolates amplified for the searched genes and some for both genes. However, other genes should be behind the detected bioactivity in non-amplifying isolates.

## Discussion

Since one of the most effective methods used in the discovery of natural products is the cultivation of new microbial strains which may represent novel important chemotypes (Fenical and Jensen, 2006; Haber and Ilan, 2013), our prime goal was to obtain bacterial isolates for screening antimicrobial activities.

The isolation of bacterial strains from *E. deficiens* after an enrichment procedure allowed the selection of a high number of fast growing heterotrophic bacteria, mainly belonging to *Proteobacteria*. Similarly, Kamke et al. (2010) also observed that one of the most abundant taxon in both libraries of *Ancorina alata* and *Polymastia* sp. was the class *Gammaproteobacteria*. *Proteobacteria* was the most abundant phylum in the phylogenetic distribution of sponge-isolated bacteria (Webster and Taylor, 2012) namely from another *Erylus* species, *E. discophorus* (Graça et al., 2013). Furthermore, species of *Firmicutes* and *Actinobacteria*, both phyla commonly present in sponges (Webster and Taylor, 2012), were also obtained.

Almost no bacterium was common among the three specimens as evidenced by the analysis of the 16S rRNA gene sequences (Figure 1). A possible explanation for this result may be the use of sponge portions from different structural parts of the sponge body that are known to be inhabited by different microbial communities (Thiel et al., 2006). The group of *Pseudoalteromonas* was the only one shared by specimens #91 and #66 which could be explained by sponges sampling site's proximity. This genus is often isolated from marine macroorganisms like sponges (Ivanova et al., 2002; Lau et al., 2005).

Several of the bioactive genera isolated in this study have been described as possessing antimicrobial activity. *Pseudoalteromonas* is a well-known bioactive compounds producer Hentschel et al., 2001; Bowman, 2007; Thomas et al., 2008; Sivasubramanian et al., 2011; Chen et al., 2012b; Vynne et al., 2012 as well as *Bacillus* (Bernal et al., 2002; Bhatta and Kapadnis, 2010; Jamal and Mudarris, 2010; Kamat and Kerkar, 2011; Kumar et al., 2012; Graça et al., 2013). Bioactivity has also been found in *Pseudomonas* (Kumar et al., 2005; Isnansetyo and Kamei, 2009; Graça et al., 2013), *Enterococcus faecalis* (Gútiez et al., 2013), *Paenibacillus* (Chung et al., 2000; Tupinambá et al., 2008; Wen et al., 2011; Graça et al., 2013) and *Microbacterium* (Lang et al., 2004; Graça et al., 2013). Antibacterial activity produced by *Psychrobacter* has been previously found (Kanagasabhapathy et al., 2008; Kennedy et al., 2009; Rojas et al., 2009; Flemer et al., 2011) but not antifungal activity. *Rhodococcus* (Chiba et al., 1999; Riedlinger et al., 2004; Lo Giudice et al., 2007; Hong et al., 2009; Abdelmohsen et al., 2010, 2014) and *Paracoccus* (Lo Giudice et al., 2007; Deng et al., 2011) showed antimicrobial activity. *Dermacoccus* was shown to produce antimicrobial, antitumor and antiprotozoal activity (Pospísil et al., 1998; Goodfellow and Fiedler, 2010). No antimicrobial activity has been detected in the genera *Cellulomonas* and *Proteus*. Okada et al. (2006) verified that the marine sponge *Erylus placenta* collected off Hachijo Island exhibited a broad spectrum of activity against several strains of yeasts and a fungus. This result is in agreement with the high levels of bioactivity against *C. albicans* obtained in our *E. deficiens* isolates.

The two preliminary and liquid assays allowed the confirmation of a great number of positive bacterial hits against *C. albicans* (72%—36 bacteria) but dissimilar results were obtained for *E. coli* and *B. subtilis*. In fact, these assays were not always coincident in the bioactivities obtained (Table 1), which could be due to differences in the cultivation (solid vs. liquid) and the extraction methods as previously observed (Bode et al., 2002; Graça et al., 2013; Haber and Ilan, 2013). In the diffusion assay there is always a potential problem with the diffusion of the bioactive molecules as well as the establishment of a concentration gradient. However, celerity and simplicity favor the use of this qualitative method (Valgas et al., 2007). Additionally, interaction between target and test microorganisms could induce a quorum-sensing response with antimicrobial production (Haber and Ilan, 2013). The assays using liquid broths have the advantage of being quantitative, simple, rapid, reproducible, and inexpensive and can be performed in a high throughput way. Furthermore, this method facilitates the upscale of the process in the case of a positive hit (avoiding constrains caused by the use of solid media).

The aqueous extracts presented higher bioactivities than the organic ones. This may be indicative of a hydrophilic nature of the bioactive molecules which could be proteins or sugars. With the acetone extraction, proteins would be precipitated and removed from the extract that could justify the decrease in bioactivity.

The genomic screening of bioactive potential is a valuable complement to the search for new bioactive molecules. The presence of PKS-I or NRPS genes was confirmed in some positive antimicrobial bacteria but non-bioactive bacteria also possessed these genes. This may indicate their ability to produce compounds with different types of activities like against other bacteria or even anticancer (Davidson et al., 2001). The PKS genes were previously found in the genera *Pseudoalteromonas* (Kennedy et al., 2009; Zhu et al., 2009; Chau et al., 2012) and *Rhodococcus* (Ayuso-Sacido and Genilloud, 2005; Abdelmohsen et al., 2014). However, to our knowledge, the present study is the first to detect PKS genes in the genera *Proteus* and *Microbacterium*. Regarding the NRPS genes, they have been previously detected in *Pseudoalteromonas* (Zhu et al., 2009; Chen et al., 2012a), *Enterococcus* (in the database: Biocyc.org^1^), *Paenibacillus* (Wu et al., 2011), *Bacillus* (Luo et al., 2014), *Rhodococcus* (Bosello et al., 2013), *Dermacoccus* (Pathom-aree et al., 2006), *Microbacterium* (Wu et al., 2012), *Citrobacter* (Siezen and Khayatt, 2008), and *Proteus* (Himpsl et al., 2010) but as far as perceived not in *Psycrobacter*, *Brevundimonas* and *Paracoccus*.

Of the confirmed bacteria that showed bioactivity against *C. albicans*, strain #91_11 belonging to the class *Gammaproteobacteria* is probably a novel species of *Pseudoalteromonas* based on the 16S rRNA gene (96.2% to *P. tetraodonis*, Figure 1). Furthermore, and based on the same gene similarity, other isolates with non-confirmed bioactivity (#66_1, #91_27, and #118_39) are also potential new taxa of *Gammaproteobacteria*.

Our results show a great antifungal potential of bacteria associated with *E. deficiens*. Curiously, in a previous work with heterotrophic bacteria of another *Erylus* species, *E. discophorus* (Graça et al., 2013), no antifungal activity was obtained. The bioactivity found was essentially against *Bacillus subtilis* which was almost absent in this work. Both works evidence for the production of bioactive compounds of bacteria associated with *Erylus* sponges which should be further studied regarding isolation and identification of the molecules accountable for the bioactivity.

## Conclusions

The isolation and phylogenetic analyses of the culturable fraction of the bacterial community isolated from *E*. *deficiens* revealed a biodiversity with putative new taxa of *Gammaproteobacteria* that should be further studied regarding genome analysis and taxonomic characterization.

A great number of *Erylus* isolates were found to produce antibacterial and high antifungal bioactivity against pathogenic and environmental strains. As far as we know, this is the first report of antimicrobial production in the genera *Cellulomonas* and *Proteus*. Furthermore, PKS and NRPS genes are potentially associated with several *Erylus* isolates, namely the genera *Proteus*, *Microbacterium* for PKS-I and *Psycrobacter*, *Brevundimonas*, and *Paracoccus* for NRPS for which these genes have not been previously described. Our results showed the biotechnological potential of bacterial diversity associated with *E*. *deficiens*, inspiring further studies for the search of new leading compounds.

### Conflict of interest statement

The authors declare that the research was conducted in the absence of any commercial or financial relationships that could be construed as a potential conflict of interest.

## Footnotes

^1^Biocyc.org.http://www.biocyc.org/EFAE749506-HMP/NEW-IMAGE?object=D-Phenylalanyl-Acps.
